# Qualitative insights from partners of individuals with Huntington’s disease: the quality of life of an under-researched population

**DOI:** 10.1186/s13023-026-04667-x

**Published:** 2026-11-10

**Authors:** Isobel Spray, Jeanette Thorpe, Ellie Johnstone, Wiebke Hannemann, Ferdinando Squitieri, Giulia Giancaterino, Paola Emilia Mina, Barbara D’Alessio, Astri Arnesen, Jiri Klempir, Olga Ulmanová, Pearl van Lonkhuizen, Anne-Wil Heemskerk, Jennifer Hoblyn, Emer O’Malley, Ramona Moldovan, Georg Bernhard Landwehrmeyer, Stephen Patrick McKenna, Alzbeta Muehlbaeck

**Affiliations:** 1 Galen Research, Manchester, UK; 2 Department of Neurology, University Hospital Ulm, Oberer Eselsberg 45, 89081 Ulm, Germany; 3 Unità Huntington e Malattie Rare Fondazione IRCCS Casa Sollievo della Sofferenza Hospital, San Giovanni Rotondo, Italy; 4 Qrare Medical Centre, Fondazione Lega Italiana Ricerca Huntington (LIRH), Rome, Italy; 5 European Huntington Association, Kristiansand, Norway; 6 Department of Neurology, Centre of Clinical Neuroscience, First Faculty of Medicine, General University Hospital, Charles University, Prague, Czechia Czechia; 7 Department of Public Health and Primary Care, Leiden University Medical Center, Leiden, Netherlands; 8 National eHealth Living Lab, Leiden University Medical Center, Leiden, Netherlands; 9 Huntington Center Topaz Overduin, Katwijk, Netherlands; 10 Trinity College Dublin, Dublin, Ireland; 11 St. John of God University Hospital, Dublin, Ireland; 12 Division of Evolution, Infection and Genomics, School of Biological Sciences, University of Manchester, Manchester, UK; 13 Manchester Centre for Genomic Medicine, St. Mary’s Hospital, Manchester University NHS Foundation Trust, Manchester, UK; 14 Department of Psychology, Babeș-Bolyai University, Cluj-Napoca, Romania; 15 Huntington Center South, Isar-Amper-Klinikum, Taufkirchen, Germany

**Keywords:** Huntington’s disease, Quality of life, Needs-based model, Partners, Caregivers, Qualitative research, Thematic analysis

## Abstract

**Objective:**

Huntington’s disease (HD) is a rare, inherited neurodegenerative disorder with profound psychosocial consequences extending beyond affected individuals to their families. This qualitative study explored the quality of life (QoL) of partners of individuals with HD, manifest (mHD) and premanifest (preHD), using a needs-based theoretical framework.

**Background:**

While the impact of HD on patients is well documented, the experiences of partners remain under-researched, particularly across different stages of disease progression. Understanding partner QoL is essential to inform holistic care in rare neurodegenerative diseases.

**Methods:**

Semi-structured interviews were conducted with adult partners or spouses of mHD or preHD individuals in the UK and Ireland (*n* = 60; UK = 48, Ireland = 12). An additional 37 interviews were conducted across Europe to assess cross-cultural consistency. Data were analysed using thematic analysis informed by the needs-based model of QoL.

**Results:**

Thematic analysis identified 376 statements from partners of mHD individuals and 491 from partners of preHD individuals. For partners of mHD individuals, key themes discussed included strained relationships, reduced communication, and loss of shared activities. Partners of preHD individuals reported preoccupation with worry, relationship difficulties, and uncertainty about the future. Findings were consistent across participating European countries, highlighting the widespread and cross-cultural nature of these challenges.

**Conclusion:**

HD significantly constrains partners’ ability to fulfil key human needs, negatively affecting QoL across disease stages. This study provides novel, theory-driven insights into an under-recognised population in rare disease research and supports the development of partner-specific QoL measures. These findings have important implications for the design and evaluation of interventions aimed at improving outcomes for families affected by HD and other rare neurodegenerative conditions.

**Supplementary Information:**

The online version contains supplementary material available at https://doi.org/10.1186/s13023-026-04667-x.

## Introduction

Huntington’s disease (HD) is a rare, inherited neurodegenerative disorder characterised by progressive degeneration of nerve cells in the brain [1]. Clinically, HD presents as a triad of motor, cognitive, and psychiatric symptoms [1], with a median survival of approximately 24 years from diagnosis [2]. The diagnosis of manifest HD (mHD) is based on the onset of clinical motor signs combined with the presence of a pathogenic CAG expansion (cytosine-adenine-guanine trinucleotide repeat sequence) in the HTT gene [1]. Premanifest HD (preHD) individuals are those who have undergone genetic testing confirming the HD gene expansion but do not yet have a clinical diagnosis [3]. Despite this, individuals with preHD may develop cognitive and behavioural changes that can precede motor onset by up to 15 years [4]. As no curative treatment currently exists, management of HD typically requires multidisciplinary care over several decades [5], placing a substantial burden not only on individuals with the disease but also on their families and partners [6]. The quality of life (QoL) of partners of individuals with HD should therefore not be overlooked.

### Partners of individuals diagnosed with mHD

As the burden of care often falls on partners of individuals with HD [6], caregiver QoL has unsurprisingly been found to be significantly correlated with patients’ functional and cognitive impairment [7]. A meta-synthesis of qualitative studies identified recurring themes in caregivers’ experiences, including struggles, impact on daily life, worries, changes in the relationship with the care recipient, and the role of social support [8]. However, a recent systematic review of QoL research in HD noted a predominance of quantitative studies, with limited qualitative evidence available [9]. Further qualitative research focusing on partner QoL is therefore essential to deepen our understanding of this subgroup’s unique experiences.

### Partners of preHD individuals

Similarly, the health-related quality of life (HRQoL) of partners of preHD gene-expansion carriers has been found to correlate with the cognitive and neuropsychiatric functioning of the preHD individual [10], and cognitive symptoms have also been associated with increased caregiver burden [11]. However, existing research largely focuses on caregiving contexts, limiting its relevance to partners of individuals who are not yet symptomatic [9]. Living with the risk and reality of HD can itself have a significant impact on relationship dynamics [12]. Partners of preHD individuals also have concerns about changes in interpersonal relationships and coping with uncertainty [13], suggesting QoL can be impacted regardless of their partners symptom status and caregiving roles. Exploring this subgroup independently, and beyond the context of caregiving, is therefore essential to capture the full complexity of partner QoL across the HD disease trajectory.

### Using the needs-based model

Whilst previous qualitative studies have identified areas of life affected by HD, few have applied a theoretical framework. Such frameworks can act as a map, guiding qualitative research from interview lines of inquiry to the interpretation and reporting of findings [14].

The needs-based model conceptualises QoL as the extent to which individuals are able to fulfil fundamental human needs [15]. When these needs are unmet, QoL is diminished. Needs may be fulfilled through satisfiers (enablers), whereas fulfilment may be prevented by inhibitors [16]. For example, socialisation can satisfy the basic need for connection and safety, but avoidance of socialisation would inhibit the fulfilment of these needs [16], thereby reducing QoL. The needs-based model can be used to describe how disease influences satisfiers and inhibitors of needs, thereby helping to explain the mechanisms through which disease affects QoL [17]. Although the model has previously been used in the context of neurodegenerative disease to measure the impact of caring for a spouse with Alzheimer’s disease [18, 19], this paper is the first to apply it to the study of QoL in partners of individuals affected by HD.

This study presents a qualitative exploration of the needs-based QoL of partners of individuals with either mHD or preHD. By applying a theoretical framework, this research provides novel insights into an under-researched population and supports the development of two partner-specific QoL measures. These measures have the potential to inform the evaluation of interventions and improve holistic care for families affected by HD.

## Methods

### Study design

This qualitative study was conducted as part of the larger multinational consortium project HEALTHE-RND. The overarching aim of the project was to develop a European eHealth care platform to support the QoL of individuals with HD and their partners. The HEALTHE-RND project also aimed to develop QoL measures for individuals diagnosed with HD, preHD gene-expansion carriers, and their partners. The present paper reports the phase of the study that explored the experiences and needs of partners of individuals with preHD and partners of those with mHD, contributing to the development of two needs-based QoL measures tailored for partners.

The consortium included five participating countries: Czechia, Germany, Italy, the Netherlands, and the UK. While this study was primarily conducted in the UK and Ireland, additional interviews were carried out by consortium partners in other countries to capture any potential cultural differences. The procedures described below relate specifically to the UK and Ireland.

### Participants

In the UK and Ireland, participants were recruited through six NHS Trusts: Birmingham, Leicester, London, Liverpool, Manchester, and Sheffield. HD centres at these sites identified eligible individuals using either locally maintained Enroll-HD databases [20], or internal clinic records. Only individuals who had previously consented to be contacted about HD research studies were approached.

Potential participants were introduced to the study by a clinician, who provided a participant information sheet and consent form. To be eligible, individuals had to be the partner or spouse of a person living with either preHD or mHD and reside in the same household. Participants were excluded if they were under 18 years of age; had serious psychiatric, neurological, sensory, or metabolic conditions; had another comorbid disorder likely to affect QoL; did not provide informed consent; had a diagnosis of mHD themselves; were carriers of the HD gene expansion; or were caring for more than one person with HD.

### Interviews

Interviews in the UK and Ireland were conducted between August 2021 and January 2023. It was anticipated that approximately 30 participants per partner group, together with the additional European data, would be sufficient for the exploration of QoL, based on previous research indicating that data saturation is typically achieved with this sample size [17, 21]. Once informed consent had been obtained, participant contact details were uploaded by individual sites to a secure electronic database. The research team accessed these details and then contacted participants to schedule interviews. Interviews were conducted by a trained researcher using interview guides tailored to each group. The interview guides were designed to elicit information from which needs-based deficits could be identified [15, 16]. Participants were first encouraged to discuss broadly how HD impacts their daily life, allowing researchers to organically explore the individuals’ need deficits, and investigate any key inhibitors stemming from HD. When necessary, researchers would use specific need facilitators that the model posits as vital for QoL as prompts for further discussion. Examples included participation in leisure activities, self-care, and relationships with family and friends.

Due to COVID-19 restrictions, all interviews were carried out remotely using secure video platforms (Zoom or Microsoft Teams). Participants were asked to be alone where possible, unless assistance was required to use the technology. Given the sensitive nature of the topic, participants were informed that they could pause or stop the interview at any time. At the start of each interview, sociodemographic information was collected to contextualise the qualitative data. The remainder of the interview focused on the participant’s personal experiences and the impact of HD on need fulfilment across different aspects of life. Each interview concluded when both the participant and interviewer agreed that all relevant issues had been sufficiently explored. Interviews were audio-recorded, and all identifying information was removed during transcription to maintain confidentiality. All procedures were conducted in accordance with Good Clinical Practice guidelines.

### Data analysis

​​Two experienced researchers, neither of whom conducted the interviews, independently analysed each transcript to minimise bias. Transcripts were analysed through the lens of the needs-based model of QoL to identify statements reflecting unmet needs. These statements were then coded, extracted from the raw transcripts, and pooled into a large dataset. The researchers were experienced in applying the needs-based model and ensured during the coding process that statements represented recognised need deficits and inhibitors. They used a qualitative, iterative assessment of intercoder consistency to encourage reflexivity [22], rather than using quantitative indicators of reliability, as this approach aligned more c​losely with the methodology of thematic analysis and its underlying epistemology. Iterative developments resulting from these qualitative assessments can be more valuable than the production of a singular reliability score [23]. Research team discussions supported nuanced judgements regarding coding consistency, relevance, and interpretation.

The wider research team then continued the thematic analysis, grouping the coded data into broader themes reflecting need-deficits. Thematic analysis followed the six steps described by Braun and Clarke [24]. Further discussions were held to ensure agreement on the final themes, subthemes, and their definitions for each subgroup. Thematic saturation had been reached when no more meaningful themes could be derived from the data.

Finally, the statements and themes derived from the UK and Irish interviews were compared with summary reports from additional interviews conducted in Czechia, Germany, Italy, and the Netherlands. The UK and Irish interviews were designated as the primary dataset because they formed the qualitative basis for item generation in the subsequent development of partner-specific quality-of-life measures. Using a single primary dataset and analytical team ensured consistency in the application of the needs-based model and in the identification of potential item content. This methodological prioritisation did not reflect an assumption that the UK and Irish participants were more representative than participants from the other European countries. Rather, the additional European interviews were used to assess the cross-cultural relevance and comprehensiveness of the themes identified in the primary analysis. Summary reports from these interviews were compared with the pooled dataset of statements to determine whether the identified themes were relevant across different cultural contexts and whether any additional themes or needs emerged that had not been identified in the initial thematic analysis.

## Results

### Demographic characteristics

A total of 60 interviews were conducted in the UK and Ireland (UK: *n* = 48; Ireland: *n* = 12), involving partners of individuals with preHD and mHD. We refer to these groups throughout as preHD partners and mHD partners.

An additional 37 interviews were conducted across Europe with participants from Czechia (*n* = 10), Germany (*n* = 8), Italy (*n* = 10), and the Netherlands (*n* = 9), to explore potential cultural differences. The demographic characteristics for these European participants are presented in Supplementary Table 1. The data presented here focus on English-speaking participants from the UK and Ireland. Their demographic characteristics are summarised in Table 1 below.

**Table 1:** Demographic characteristics of partners of individuals with mHD and preHD in Ireland and the UK

Participants in total (*N* = 60)		
	mHD partners (*n* = 31)	preHD partners (*n* = 29)
**Age: (years**)		
Mean (SD)	58.3 (9.4)	46.1 (10.7)
Range	38–75	30–69
**Sex: n (%)**		
Male	16 (51.6)	16 (55.2)
Female	15 (48.4)	13 (44.8)
**Ethnicity: n (%)**		
Caucasian	31 (100.0)	27 (93.1)
Hispanic or Latino Origin	0 (0.0)	1 (3.4)
Mixed	0 (0.0)	1 (3.4)
**Living with: n (%)**		
Partner Only	21 (67.7)	13 (44.8)
Children aged under 18 years	4 (12.9)	9 (31.0)
Children aged 18 years and older	5 (16.1)	5 (17.2)
Children in both age groups (under and over ​18)	1 (3.2)	2 (6.9)
**Employment status: n (%)**		
Full-time	10 (32.3)	23 (79.3)
Part-time	9 (29.0)	1 (3.4)
Unemployed	1 (3.2)	1 (3.4)
Retired	7 (22.6)	1 (3.4)
Working in the home	3 (9.7)	1 (3.4)
College	0 (0.0)	1 (3.4)
Sick leave	1 (3.2)	1 (3.4)
**Self-perceived general health: n (%)**		
Poor	0 (0.0)	0 (0.0)
Fair	8 (25.8)	3 (10.3)
Good	14 (45.2)	16 (55.2)
Very Good	9 (29.0)	10 (34.5)
**preHD Partners: Duration since genetic confirmation (years)**: ***n*(%)**		
≤2	-	5 (17.2)
3 to 5	-	6 (20.7)
6 to 10	-	7 (24.1)
11 to 15	-	8 (27.6)
≤​16	-	3 (10.3)
**mHD Partners: Duration of symptoms (years)**, ***n*(%)**		
≤ 2	8 (25.8)	-
3 to 5	8 (25.8)	-
6 to 10	8 (25.8)	-
11 to 15	3 (9.7)	-
≤​​ 16	4 (12.9)	-
**mHD Partners: Partner is: n (%)**		
Aggressive	8 (25.8)	-
Prone to wandering	1 (3.2)	-
Incontinent	9 (29.0)	-
Unable to be left alone	10 (32.3)	-
Unable to recognise their partner	4 (12.9)	-
None of the above	12 (38.7)	-

Note: N: total number of participants; n: number of participants in subgroup, SD: Standard Deviation; HD (Huntington´s Disease), mHD Partners (partners of individuals with manifest HD) preHD (partners of individuals with premanifest HD)

### Thematic analysis of interviews with mHD partners

**Fig. 1:**
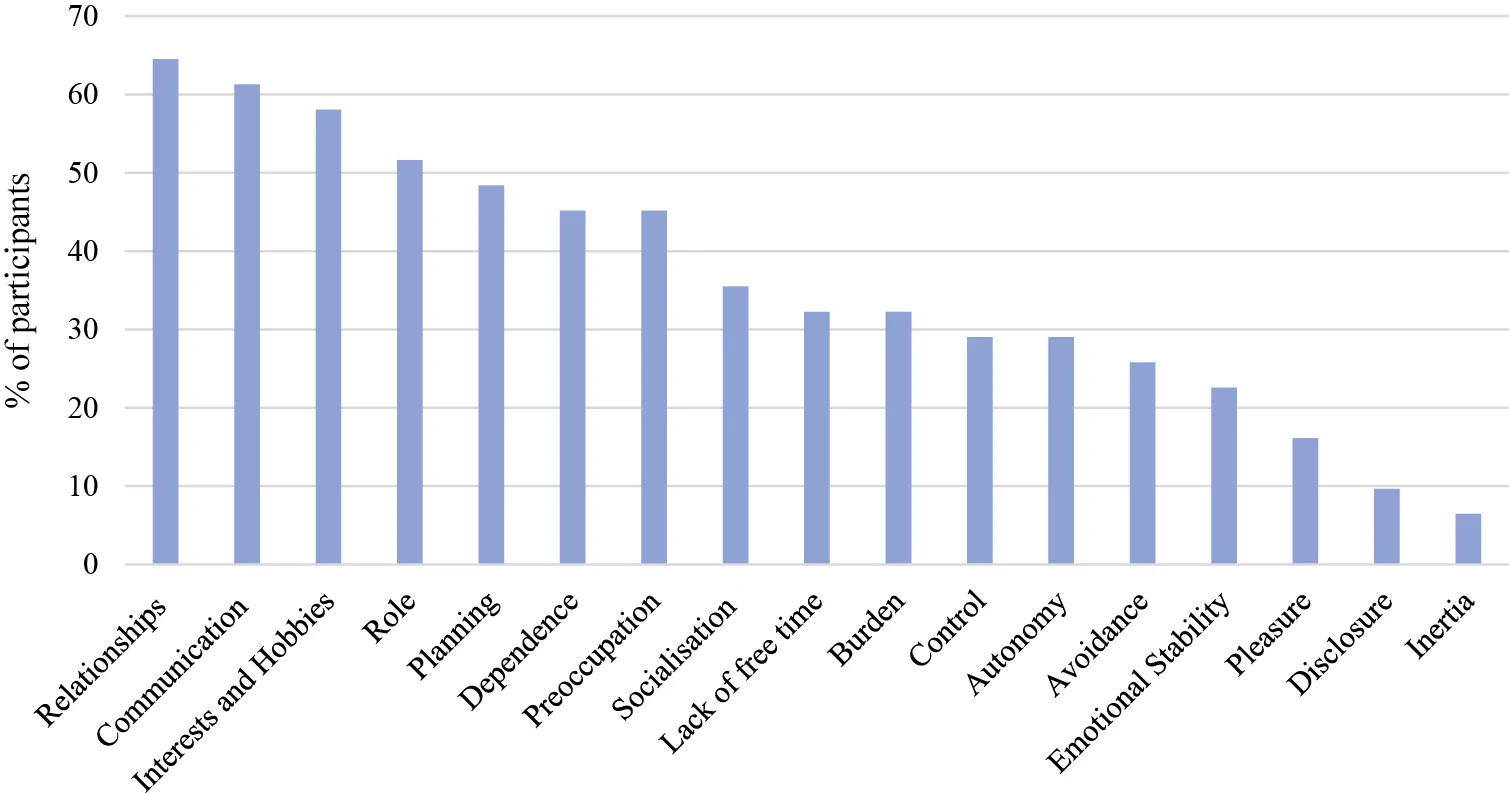
Main themes among partners of individuals with mHD

Drawing on the needs-based model of QoL, a total of 376 statements reflecting unmet needs were extracted from interviews with mHD partners. These statements were analysed using thematic analysis, resulting in the identification of 17 distinct themes representing perceived need deficits and barriers to need fulfilment (Fig. 1).

**Fig. 2:**
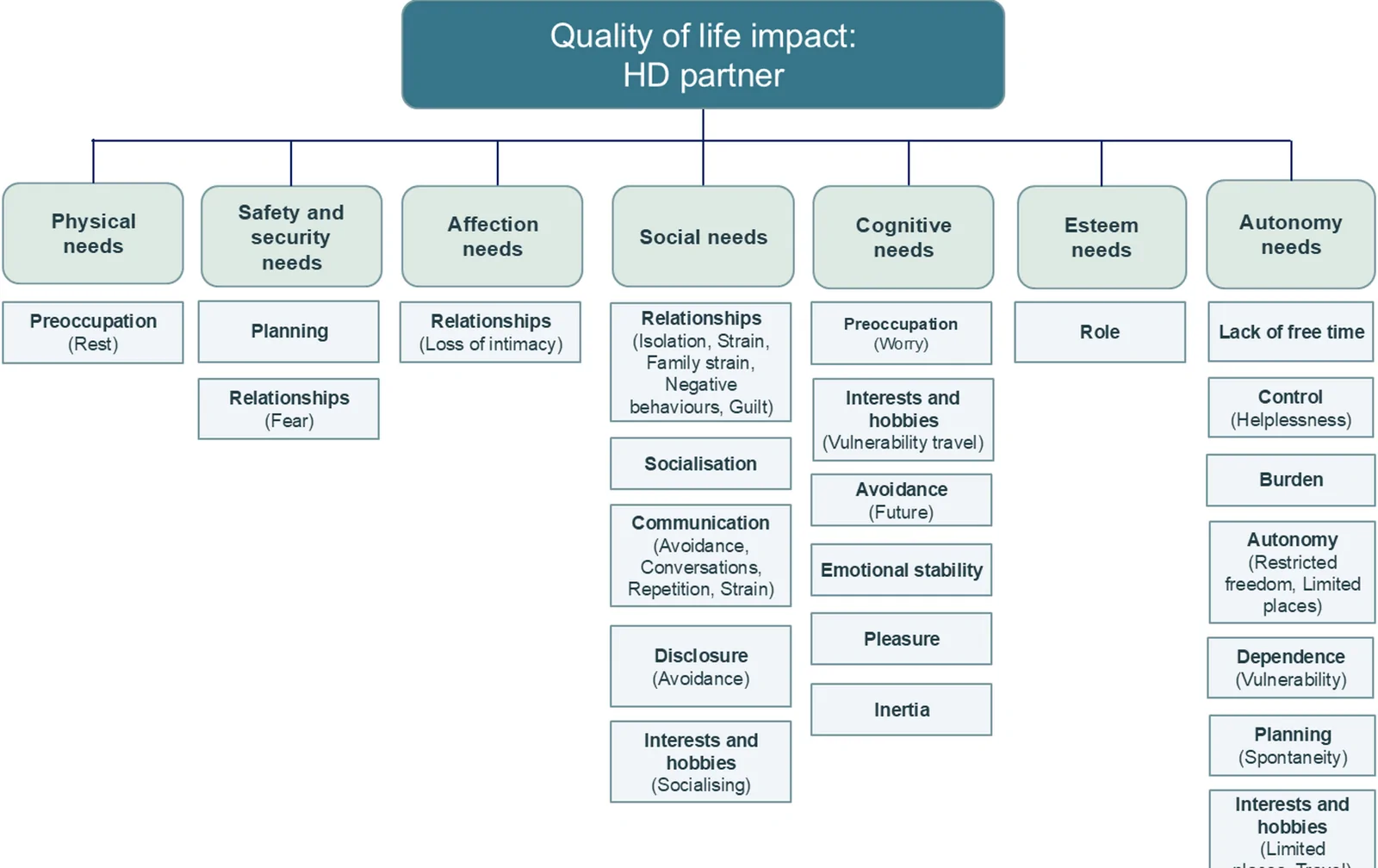
Main themes and associated subthemes from interviews with partners of individuals with mHD categorised by need

The main themes, along with their subthemes in brackets, are presented in Fig. 2. This figure illustrates how each theme maps onto core needs in the needs-based model of QoL. Some themes appear under more than one need, due to differences between subthemes. Such variation is expected, as inhibitors can impact multiple needs simultaneously.

Two additional categories - ‘Coping Mechanisms’ and ‘Stigma’- were identified during analysis but did not fit with the needs-based model. *Coping Mechanisms* were excluded due to their highly individualised nature, which rendered them unsuitable for categorisation within a shared thematic structure. *Stigma* was excluded as participants’ accounts referred primarily to others’ perceptions of HD, rather than the direct psychological impact on themselves.

The eight most frequently discussed themes are explored in greater depth below, with illustrative quotations provided:

#### Relationships

HD placed considerable strain on interpersonal relationships. Many partners described substantial changes in relationship dynamics, often linked to personality changes in the individual with HD. One participant reflected, “It’s like living with a completely different person” (Female, 47 years).

In addition to spousal relationships, several participants described a lack of understanding or awareness of HD among extended family members, contributing to feelings of isolation. One participant expressed this sense of estrangement: “Some of my family don’t even understand it really either… I don’t even talk to them anymore” (Female, 48 years). These accounts suggest that the relational burden of HD extends beyond the immediate caregiving relationship, often exacerbated by social and familial disengagement.

#### Communication

Difficulties with open communication were frequently discussed. Many participants reported that conversations about emotionally sensitive topics, particularly those relating to disease progression, were often avoided. One participant reflected: “It would probably be really upsetting for both of us, I think, to talk about it [talking about feelings]” (Female, 48 years).

For others, the communication challenges arose from the neurological and cognitive symptoms of HD itself, leaving partners feeling isolated and emotionally unsupported: “The worst is when she’s not able to communicate, you’ve no-one to talk to at the end of the day” (Male, 45 years).

In some cases, communication was deliberately curtailed to avoid potential conflict or agitation: “We don’t bring up any, you know, subjects that we know will trigger him… So you can’t get into a debate with [name] like about politics or – unless you’re agreeing with him” (Female, 57 years).

#### Interests and hobbies

Engagement in personal interests and recreational activities was substantially reduced for many participants, largely due to caregiving responsibilities associated with HD. Activities were frequently deprioritised due to time constraints and emotional fatigue: “I’ve kind of… given up on my hobbies because I’ve found it very difficult to commit to things” (Female, 61 years). Others noted that hobbies shared with their partner were no longer possible to engage in due to HD, representing a disruption to both social and pleasure-based needs, “We used to go to the cinema, not a lot, but we enjoyed the cinema… we haven’t been able to do that in the last three or four years. So that’s a sadness, really, I think that’s something I miss” (Male, 72 years).

#### Role

Shifts in household and caregiving roles emerged as a prominent theme among participants, with many describing a redistribution of domestic responsibilities as their partner’s condition progressed. As one participant reflected: “I now clean the house, do the ironing, do the shopping… I had to pick up the housewife chores as well as the work chores” (Male, 64 years).

Beyond the practical aspects, several interviewees expressed a sense of loss regarding their role as a spouse, as caregiving responsibilities came to dominate the relationship dynamic: “I feel more of a carer than a wife” (Female, 38 years).

#### Planning

Loss of spontaneity in daily life was frequently cited as a significant challenge by partners of individuals with mHD. Many participants described how constant logistical preparation constrained their autonomy and flexibility, “You can’t be spontaneous like you used to be. You’ve got to plan if you are going out” (Female, 61 years). Therefore, planning was a key responsibility for partners: “Always thinking about how you get there. You’re not just walking yourself; you’re not just moving yourself, you’ve got to move them as well” (Male, 61 years).

#### Dependence

Participants frequently described an increased sense of responsibility for their partner’s physical and emotional care. The reliance of the mHD individuals on their partners often had a profound impact on the partner’s autonomy. One participant detailed the extent of care required: “I do everything for her… just like throughout the day, it’s everything, from having…from her toileting to her washing, to feeding, to moving about, to getting her drinks, she can’t…she does virtually nothing for herself anymore” (Male, 62 years).

In addition to performing daily care tasks, participants noted that their routines were shaped by the need to be constantly present to ensure their partner’s safety: “He sits and watches telly all day…I have to be here because of his balance and coordination issues” (Female, 61 years).

#### Preoccupation

Many partners described persistent thoughts and fears about their partner’s safety. This constant vigilance manifested as heightened concern and ongoing mental engagement, reflecting the emotional burden of caregiving. One participant described taking active measures to monitor their partner throughout the day: “I have cameras around the house. So, I check in on the cameras, a few times a day“ (Male, 50 years).

Others acknowledged that the preoccupation with thoughts about their partner was particularly problematic at night, resulting in an impact on the physiological need for rest: “Just think about things over and over again in our head… it’s hard to switch off, whereas in the daytime you’ve got something that occupies your mind… But at night time you mull it over, yeah…but night time is worse, definitely” (Female, 38 years).

#### Socialisation

The impact of HD on socialising was another prominent theme, with many participants reporting a significant reduction in social interactions, often attributed to the increased caregiving responsibilities. One participant highlighted the absence of a social life: “Zero social life…only people we ever see are when my children come round. But, yeah, zero friends, zero social life. I just do this“ (Male, 62 years). Another participant explained: “I can’t leave [husband] in the evenings. So, somebody says can you come out? Do you want a… I have to say no” (Female, 61 years).

Some participants noted how their partners’ behaviour made it difficult for them to socialise and connect with others, “Because he fell out with all of them [friends]…I couldn’t socialise as couples with them anymore” (Female, 46 years).

Additionally, the social anxieties of some mHD individuals presented barriers to participants’ social engagement: “She doesn’t like people seeing her… so then that stops us then from going out anywhere” (Female, 48 years).

### Thematic analysis of interviews with preHD partners

**Fig. 3:**
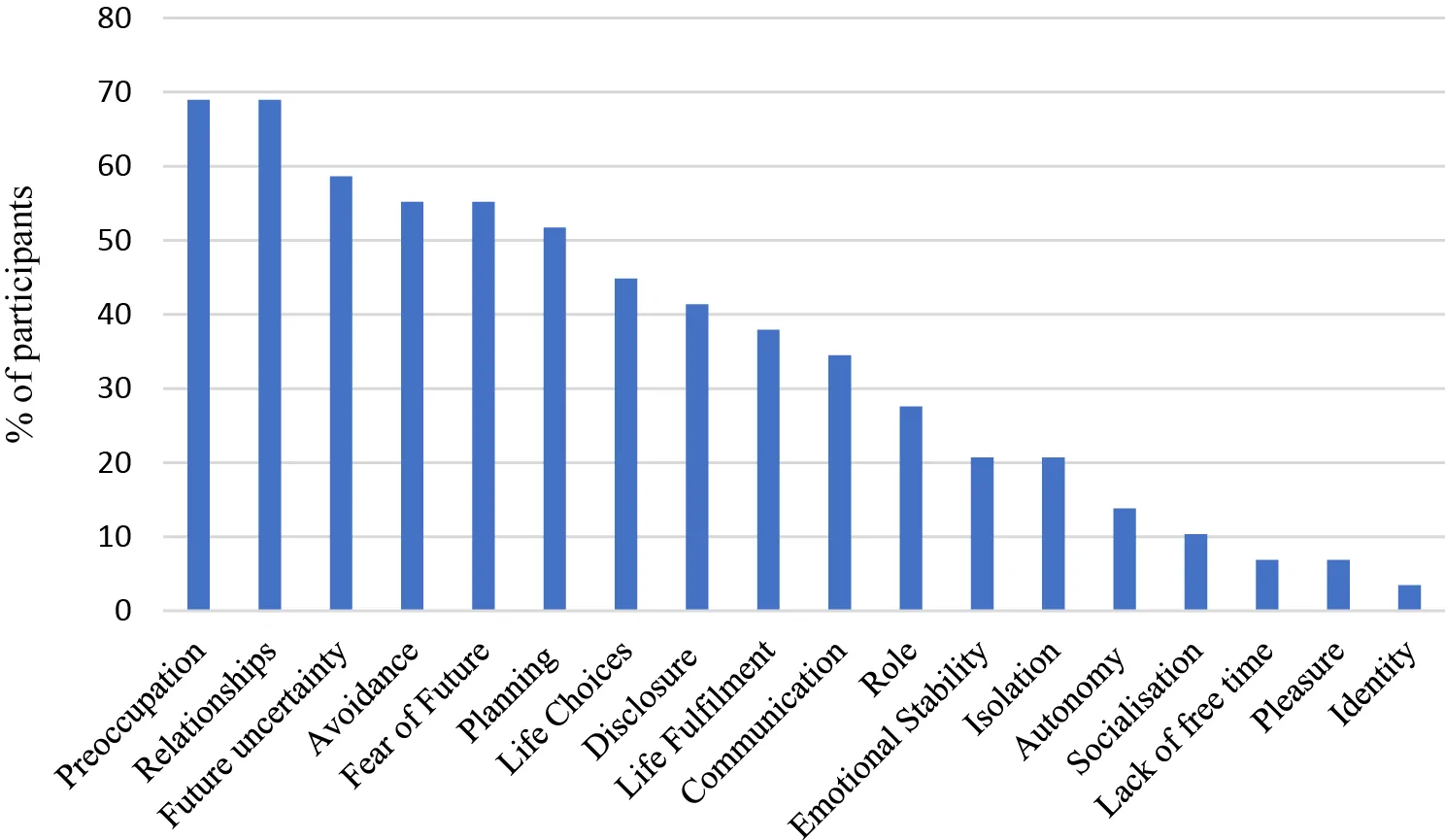
Main themes from interviews with partners of preHD individuals

Transcripts were analysed using thematic analysis, resulting in the identification of 18 distinct themes from 491 statements, representing perceived need deficits and barriers to need fulfilment (Fig. 3). The themes most frequently discussed by participants were “Preoccupation” and “Relationships,” with each being addressed by 20 participants.

Two other themes, “Coping Mechanisms” and “Control,” emerged from the data but were not included in the final analysis due to their divergence from the needs-based model. Specifically, *Coping Mechanisms* was excluded because it was highly individualised and context-dependent, while *Control* was not deemed relevant to the preHD stage, as it was discussed in relation to a potential future caregiving role rather than current experiences.

**Fig. 4:**
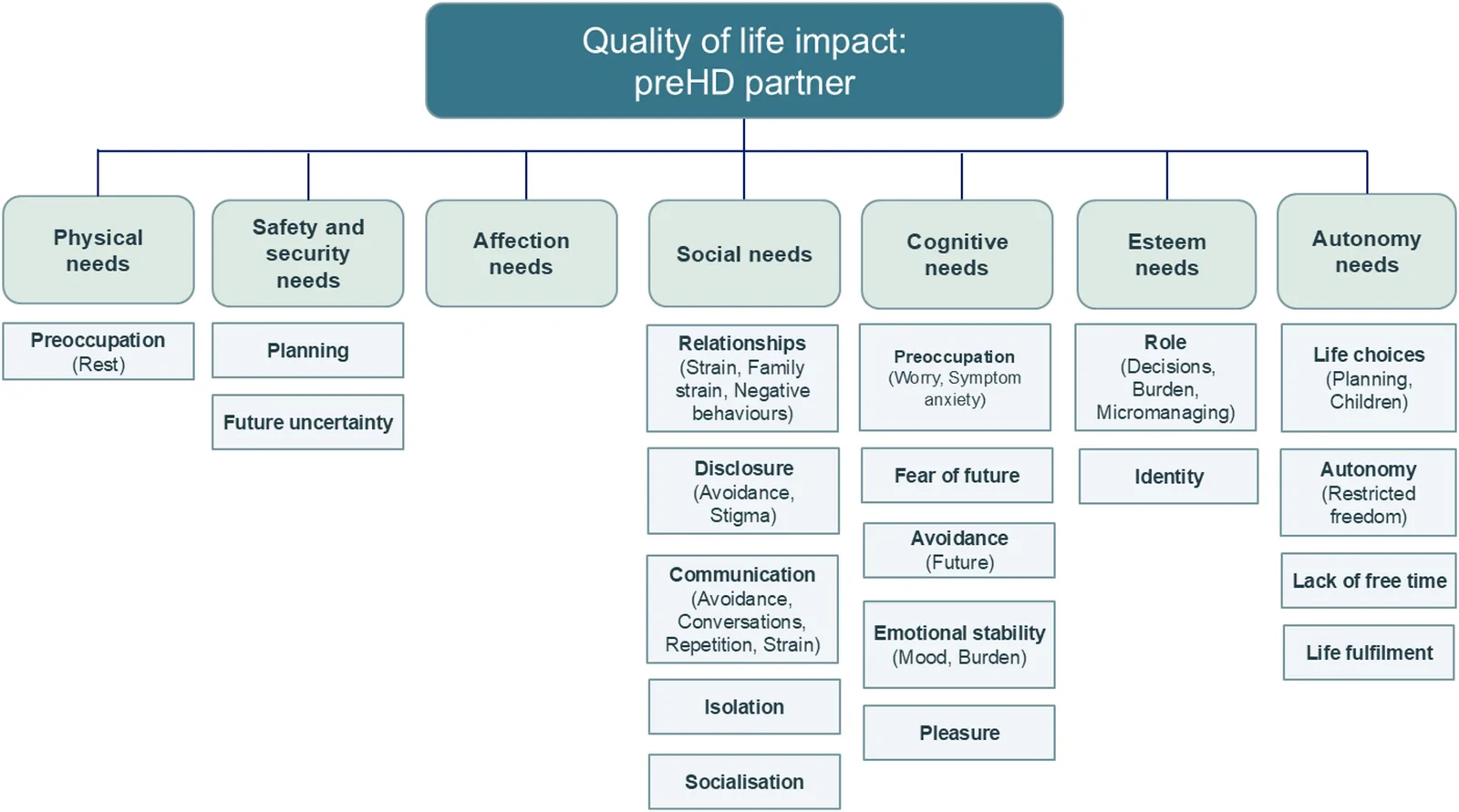
Main themes and associated subthemes from interviews with partners of preHD individuals categorised by need

The main themes and corresponding subthemes obtained from the interviews are presented in Fig. 4, reflecting the nuanced dimensions of participants’ experiences. This figure also illustrates how each theme maps onto core needs in the needs-based model of QoL.

The eight most frequently discussed themes are explored in greater depth below, with illustrative quotations provided to contextualise the findings.

#### Preoccupation

Participants frequently described preoccupation with HD-related thoughts. For many, this manifested as heightened symptom anxiety, with participants exhibiting a significant level of vigilance regarding potential signs of HD in their partners: “I will make connections that even he doesn’t make that no one else makes, and I’ll be like, oh my gosh, is that something to do with HD? … I’ll say stuff. And I’ll even say I’m sorry, I’m doing that thing that I do” (Female, 44 years). Such an all-encompassing preoccupation is a significant drain on mental energy, inhibiting cognitive resource needs.

#### Relationships

For many preHD partners, despite the absence of a clinical diagnosis of mHD, their partners exhibited cognitive changes that impacted need fulfilment within their relationships. Participants described conflicting emotions, balancing empathy for their partner with feelings of hurt: “It’s hard not to [get hurt by outbursts] … but at the same time there’s a little voice in the back of your head constantly saying it’s not her fault” (Male, 51 years).

Participants also reported strain within wider family relationships. One participant emphasised the importance of shielding their child from relationship tensions, stating that “we need to try our very best not to argue in front of her [daughter] and have a positive family life… rather than a negative one” (Male, 40 years).

#### Future uncertainty

Future uncertainty was discussed in relation to a struggle with the security of their future in a practical sense, due to the unknowns that come with an HD diagnosis. For some participants, this involved discussions about future caregiving:” I have actually thought in the past, oh god will I have to care for [male name]? … I need to make that decision [whether to care for him] now or, you know, I don’t know…” (Female, 30 years).

Others discussed uncertainty related to finances, “my main worry for the future is because [name]’s so good at burning through all our money… what happens when I have to stop work?” (Male, 51 years).

#### Avoidance

Some participants described actively avoiding future-related thoughts, as one participant explained, “I think it’s probably best if we just concentrate on what’s happening today… because our future, we have no idea where that’s going to go” (Female, 47 years).

Others suggested that this avoidance was a deliberate attempt to manage distress, as thoughts about HD often triggered difficult emotions. One participant noted that “it’s just something that I avoid because it’s too painful” (Female, 32 years).

#### Fear of future

Whilst future uncertainty led to discussions of the practical concerns of dealing with the unknown for some participants, it was important to make a distinction between these considerations, and a more all-encompassing fear of the inevitable changes HD will bring to the lives of partners. This theme can be defined by the intense emotional impact of anxiety and sadness, inhibiting the cognitive need for emotional stability, as opposed to future security. As one participant reflected, “one of the things that gets me at the moment is seeing… older couples walking down the street… then thinking that that’s not going to be how it’s going to be for us. And that really gets me” (Female, 32 years).

Others described struggling with the certainty of disease onset, rather than uncertainty about how it would manifest. This often prevented them from engaging in typical life milestones, such as celebrating birthdays, due to their association with disease progression. One participant explained, “I think birthdays are quite hard… because it’s an extra year… I’d think there’s only now nine more years… that was a constant thing in my head… it’s like a clock in your head constantly” (Male, 32 years).

#### Planning

Planning for future decisions was a key consideration for many participants, to ensure security needs were fulfilled. For some, this included end-of-life planning, even prior to the onset of their partner’s symptoms, as one participant explained: “Do you think we should keep a record now… like a living will… of how he’d want to be treated [talking about euthanasia]” (Female, 30 years).

Others described the burden of having to plan. In this case, the participant planned to increase their workload to account for the impact HD will have in the future “I think that, sort of, consciously, and subconsciously made me think, well if I work really hard, you know, I can make us financially really stable and we can enjoy ourselves … make the most of it. But that is like quite a lot of…only until recently I’ve noticed, that put a hell of a lot of pressure on me personally” (Male, 32 years).

#### Life choices

A number of participants described how their partner’s HD genetic status significantly influenced major life decisions, including those relating to careers, relocation, and marriage. One participant explained how HD influenced their choice of career, “I knew that I wanted to have a job that paid quite well… that I could dip in and out of if I needed to… so if [name] became ill and I needed to take a step back, then I know I could do that” (Female, 49 years).

Others discussed how reproductive decisions were directly affected by the potential transmission of the HD gene. One participant reflected on the ethical complexities of these life choices, “we briefly discussed having a child, but it just wasn’t an option… if it does carry the gene, you’re urged to terminate… and we couldn’t do that, knowing there’s a 50/50 chance we might have to terminate” (Male, 30 years). 

#### Disclosure

Many participants described avoiding disclosure of their partner’s condition. Some chose to withhold this information due to concerns about how others might respond, particularly fears of pity or altered perceptions. As one participant explained, “I don’t want them to know that my children might have it, just so there’s no pity… I don’t want to talk about it, basically. It’s a situation I don’t want to talk about” (Female, 32 years).However, disclosure was also discussed in relation to a lack of understanding of HD among others, describing how limited awareness of the disease, particularly its cognitive and behavioural symptoms, could be frustrating and hurtful. As one participant noted, “I think that’s the cruelty of this particular disease… when people go, ‘oh, is that the movement thing?’… actually your whole personality can change” (Female, 47 years). These experiences suggest that both disclosure and non-disclosure may inhibit the fulfilment of social connection needs. 

#### Transcultural comparison

A supplementary set of 37 interviews conducted across four European countries, Czechia (n = 10), Germany (n = 8), Italy (n = 10), and the Netherlands (n = 9) provided cross-cultural validation of the core findings. The thematic patterns identified among both preHD and mHD partners in these countries mirrored those found in the UK and Ireland, supporting the broader applicability of the needs-based model across diverse sociocultural contexts. All themes that arose from these interviews, as identified using reflexive thematic analysis by researchers in their respective countries, were collated and compared with the findings from the UK and Ireland to look for any distinctive differences. Themes were either already clearly consistent with those found in the UK and Ireland, or could conceptually be considered equivalent. For example, a theme titled ‘Not talking about the disease’ in the Netherlands was considered equivalent to ‘Communication, Avoidance’. The most frequently discussed themes across all countries and partner groups were ‘Life choices’ and ‘Future uncertainty’. While the overarching themes remained consistent, subtle cultural differences were noted in how certain needs were experienced or expressed. For example, variations in the availability of support structures in the context of the overarching theme of ‘Lack of free time’: “There is day care, there is night care, which the health insurance companies also cover, but if you don't get a place, then it's no use to me. If everything is available in theory and nothing is available in practice” (Female, 58 years). These findings suggest that while the psychosocial impact of HD on partners is universally experienced, cultural context does not change the nature of these needs but instead may influence how they are communicated or addressed.

## Discussion

The findings of this study provide important insights into the QoL of individuals whose partners are either carriers of the HD gene expansion (preHD) or have received a diagnosis of manifest HD (mHD). Drawing on the needs-based model of QoL, participants described a range of challenges associated with their partner’s condition. They highlighted how the presence of the HD gene expansion, whether their partner was symptomatic or not, can constrain personal need fulfilment. These findings highlight the substantial emotional and practical burden experienced by partners across the disease trajectory. Furthermore, the inclusion of interviews from Czechia, Germany, Italy, and the Netherlands supports the broader applicability of these findings across diverse cultural and healthcare contexts. Previous research has identified similar themes, including autonomy [25], role change, relationship dynamics, and preoccupation as salient themes affecting QoL [6, 8]. However, these studies have not distinguished between the experiences of partners of individuals with preHD and partners of those with mHD, despite the likelihood that different stages of disease progression will uniquely shape partners’ experiences [26].

Differences between groups were particularly evident in relation to relationships, with caregiving responsibilities shaping relationship dynamics and contributing to significant challenges for mHD partners [6, 8, 27]. The needs-based model conceptualises relationships as key enablers for needs such as affection and security, and relationships are commonly reported across different chronic disease groups in studies of needs-based QoL [21, 28, 29]. The current study found that HD disrupted the ability to maintain fulfilling romantic connections for mHD partners, often due to the behavioural and personality changes in mHD individuals and the associated relationship strain. From a needs-based perspective, such relational challenges appear to inhibit the fulfilment of core psychological needs, particularly those related to affection and security needs that are typically met within intimate partnerships. Emerging literature increasingly acknowledges the centrality of these needs for maintaining not only QoL, but broader psychological and physical health [30, 31].

Partners of individuals with preHD, however, primarily struggled with the emotional burden of anticipating behavioural changes and grappling with implications for the future of their relationship and family life. From a needs-based perspective, this relationship uncertainty may inhibit fulfilment of the need for safety and security, argued by many to be one of the most fundamental human needs as it spans not only physiological safety [32] but social safety [33]. These findings therefore suggest that while HD inhibits need fulfilment for both groups, the mechanisms through which HD impacts QoL, and the types of needs inhibited, differ between groups. Findings from both, however, are consistent with literature on partners of those with neurodegenerative diseases, which indicates that neurodegenerative disease can have complex effects on relationships [34, 35].

Avoidance emerged as a shared theme across both partner groups, though it was discussed less frequently by partners of individuals with mHD, suggesting avoidance may become less feasible as the disease progresses. Lowit & Van Teijlingen found that for mHD partners, avoidance was expressed in terms of avoiding familial discussions about the disease [36]. However, in this analysis, avoidance was subsumed under the broader theme of communication, which emerged as a prominent theme for the mHD subgroup.

In contrast, avoidance was more commonly reported by preHD partners. Consistent with prior findings [36] the preHD partners in this study described efforts to ignore early signs and symptoms of HD to maintain emotional stability and avoid confronting distressing realities associated with the disease. In the current study, avoidance behaviours were commonly used as a strategy to continue daily life without having to confront the difficult emotions that HD could trigger. However, avoidance may inhibit authentic emotional expression and thereby interfere with the need for connection. A link between experiential avoidance and lower QoL has been found in the context of other disease groups [37, 38] providing support for interventions such as acceptance and commitment therapy (ACT), which specifically targets experiential avoidance. More recently, studies have assessed the value of ACT for carers of individuals with HD [39, 40]. Our findings suggest that such approaches may also be relevant for preHD partners, rather than being reserved for partners who have already assumed a more explicit caregiving role. In clinical practice, healthcare professionals could therefore explore avoidance and difficulties discussing HD during routine consultations with partners, including during the premanifest stage, and offer referral to psychological support where these difficulties are affecting emotional wellbeing, relationships, or daily functioning [41]. The overall aim of psychological interventions is to improve QoL for individuals with HD and their partners by providing education and developing effective coping strategies to manage disease-related psychosocial stressors. Such interventions could focus on enhancing psychological flexibility, supporting the management of difficult thoughts and emotions related to HD, and helping partners to maintain valued relationships, activities, and social roles [41, 42]. From a clinical perspective, these findings suggest several opportunities for more proactive support of partners throughout the HD disease course. Partners should not only be approached once substantial caregiving responsibilities have developed; clinicians could periodically enquire about their emotional wellbeing, avoidance of HD-related concerns, changes in relationships and social connections, and their ability to maintain valued interests and activities.

While some findings ​were consistent with prior literature, the role of interests and hobbies in HD partner QoL is​ a novel finding. Interests and hobbies are theorised to contribute to QoL,​ through benefits to self esteem and the relationship between hobbies, social connection and identity[43]. The needs-based model therefore positions interests and hobbies as satisfiers. While commented upon by many mHD partners, it was not however a theme amongst the preHD partners, suggesting it could be related to caring commitments and ability to continue shared interests.

## Limitations

Future studies may benefit from further stratifying partner subgroups, refraining from a purely dichotomous approach to sampling. Although this study included both preHD and mHD partner groups, variability in disease expression is likely to influence partner experiences within these groups. It could be particularly beneficial to differentiate between preHD partners whose partners are symptomatic with cognitive symptoms and those without symptoms. We noted in the current study within the ‘Relationships’ theme, QoL issues differed slightly dependent on whether partners were symptomatic or not. When preHD partners had asymptomatic partners, issues discussed often focused on the strain HD had on their family due to complexities surrounding the genetic test results. However, preHD partners who had partners with some psychiatric symptoms expressed how such symptoms influenced social interactions in their relationship.

Due to the breadth of themes and the volume of qualitative data generated, the present analysis was limited in the depth with which all findings could be explored. Although the eight most frequently reported themes for each group were discussed, less frequently mentioned themes remain important and will contribute to the development of QoL measurement items. It should also be noted that the primary aim of this research was to inform the development of needs-based QoL measures, and therefore both interviews and analysis focused on areas of unmet need. The relative absence of positive accounts reflects this focus on QoL deficits, consistent with the aims of the HEALTHE-RND project. As with all self-reported interview data, the possibility of social desirability bias cannot be excluded. However, the use of confidential interviews conducted by researchers independent of participants’ personal and clinical relationships may have facilitated more open discussion of sensitive experiences. Future research should explore how needs are fulfilled and how satisfiers positively contribute to QoL, as these aspects may provide a more comprehensive understanding of partner experiences.

## Conclusions

This study highlights how HD affects need fulfilment among partners. This study adds a unique contribution to the literature on QoL in HD, by exploring in depth the QoL of partners at differing stages of the disease progression using a meaningful outcome model, exploring QoL beyond general health and partner symptoms.

The findings illuminate to clinicians the importance of taking a holistic approach to treatments and interventions for HD, highlighting the impact the disease can have on the QoL of those close to patients as well as the patients themselves. The findings emphasise the importance of support for partners of individuals with HD.

Using the needs-based model also allowed this qualitative work to form the basis for the development of two unidimensional partner QoL measures. The measures will be beneficial in the assessment of partner-focused interventions, and assessment of the impact of patient-centred interventions on partner QoL. When combined with the needs-based patient measures, they will allow for a holistic assessment of the impact of HD across the disease spectrum, valuable to incorporate both in large scale registries and clinical settings, reflecting meaningful changes to QoL.

## Supplementary Information

Below is the link to the electronic supplementary material.

Supplementary Material 1

## Data Availability

The raw data supporting the conclusions of this article will be made available by the authors, without undue reservation, upon reasonable request.
