# Supplementary material for: Qualitative insights from partners of individuals with Huntington’s disease: the quality of life of an under-researched population
Source: Orphanet J Rare Dis. 2026 Nov 10;21:405. doi: 10.1186/s13023-026-04667-x (PMC13753496; doi:10.1186/s13023-026-04667-x)
Supplement: Supplementary file 1 [file 13023_2026_4667_MOESM1_ESM.docx]

# Supplementary material

## Supplementary table 1

*Demographic characteristics of participants from supplementary interviews conducted in Czechia, Italy, Germany, and the Netherlands*

| **Participants in total (N=37)** | | |
| --- | --- | --- |
|  | **mHD partners (n=20)** | **preHD partners (n=17)** |
| **Age: (years**) |  |  |
| Mean (SD) | 50.1 (11.1) | 37.8 (9.6) |
| Range | 29-65 | 25-58 |
| **Sex: n (%)** |  |  |
| Male | 10 (50.0) | 7 (41.2) |
| Female | 10 (50.0) | 10 (58.8) |
| **Ethnicity: n (%)** |  |  |
| Caucasian | 20 (100.0) | 16 (94.1) |
| Mixed | 0 (0.0) | 1 (5.9) |
| **Living with: n (%)** |  |  |
| Partner Only | 18 (90.0) | 14 (82.4) |
| Children under 18 | 0 (0.0) | 2 (11.8) |
| Children over 18 | 2 (10.0) | 1 (5.9) |
| **Employment status: n (%)** |  |  |
| Full-time | 12 (60.0) | 10 (58.8) |
| Part-time | 5 (25.0) | 4 (23.5) |
| Unemployed | 3 (15.0) | 2 (11.8) |
| Self-employed | 0 (0.0) | 1 (5.9) |
| **Self-perceived general health: n (%)** |  |  |
| Poor | 0 (0.0) | 0 (0.0) |
| Fair | 4 (20.0) | 1 (5.9) |
| Good | 14 (70.0) | 10 (58.8) |
| Very Good | 2(10.0) | 6 (35.3) |
| **preHD Partners: Duration since genetic confirmation (years): n (%)** | | |
| <2 | - | 4 (25.0) |
| 3 to 5 | - | 9 (52.9) |
| 6 to 10 | - | 3 (17.6) |
| 11 to 15 | - | 0 (0.0) |
| <16 | - | 0 (0.0) |
| **mHD Partners: Duration of symptoms (years), n (%)** | | |
| <2 | 1 (5.0) | - |
| 3 to 5 | 6 (30.0) | - |
| 6 to 10 | 8 (40.0) | - |
| 11 to 15 | 3 (15.0) | - |
| <16 | 2 (10.0) | - |
| **mHD Partners: ,,Partner is:’’, n (%)** |  |  |
| Aggressive | 8 (25.8) | - |
| Prone to wandering | 1 (3.2) | - |
| Incontinent | 9 (29.0) | - |
| Unable to be left alone | 10 (32.3) | - |
| Unable to recognise their partner | 4 (12.9) | - |
| None of the above | 12 (38.7) | - |

Note: N: total number of participants; n: number of participants in subgroup, SD: Standard Deviation; HD (Huntington´s Disease), mHD Partners (partners of individuals with manifest HD) preHD (partners of individuals with premanifest HD).
